# Factors associated with malaria vaccine uptake in Nsanje district, Malawi

**DOI:** 10.1186/s12936-024-04938-7

**Published:** 2024-04-17

**Authors:** Atusaye J. Simbeye, Save Kumwenda, Lauren M. Cohee, Dickens Omondi, Peninah K. Masibo, Hesborn Wao, Shehu S. Awandu

**Affiliations:** 1 Department of Biomedical Sciences, School of Health Sciences, Jaramogi Oginga Odinga University of Science and Technology, P. O. Box 210-40601, Bondo, Kenya; 2 Department of Public and Environmental Health Sciences, School of Science and Technology, Malawi University of Business and Applied Sciences, Chichiri, Private Bag 303, Blantyre, Malawi; 3 Department of Pediatrics, Division of Infectious Disease and Tropical Pediatrics, Center for Vaccine Development and Global Health, University of Maryland School of Medicine, 655 B Baltimore St S, Baltimore, MD 21201 USA; 4 Department of Clinical Sciences, Liverpool School of Tropical Medicine, Pembroke Place, Liverpool, L3 5QA UK; 5 School of Public Health, Amref International University, P. O. Box 27691-00506, Nairobi, Kenya; 6 African Population and Health Research Centre (APHRC), P. O. Box 10787-00100, Nairobi, Kenya

**Keywords:** Malaria vaccine, Malawi, Vaccine uptake, Vaccine coverage, Factors influencing vaccine uptake, RTS,S

## Abstract

**Background:**

Malaria remains a significant global health burden affecting millions of people, children under 5 years and pregnant women being most vulnerable. In 2019, the World Health Organization (WHO) endorsed the introduction of RTS,S/AS01 malaria vaccine as Phase IV implementation evaluation in three countries: Malawi, Kenya and Ghana. Acceptability and factors influencing vaccination coverage in implementing areas is relatively unknown. In Malawi, only 60% of children were fully immunized with malaria vaccine in Nsanje district in 2021, which is below 80% WHO target. This study aimed at exploring factors influencing uptake of malaria vaccine and identify approaches to increase vaccination.

**Methods:**

In a cross-sectional study conducted in April–May, 2023, 410 mothers/caregivers with children aged 24–36 months were selected by stratified random sampling and interviewed using a structured questionnaire. Vaccination data was collected from health passports, for those without health passports, data was collected using recall history. Regression analyses were used to test association between independent variables and full uptake of malaria vaccine.

**Results:**

Uptake of malaria vaccine was 90.5% for dose 1, but reduced to 87.6%, 69.5% and 41.2% for dose 2, 3, and 4 respectively. Children of caregivers with secondary or upper education and those who attended antenatal clinic four times or more had increased odds of full uptake of malaria vaccine [OR: 2.43, 95%CI 1.08–6.51 and OR: 1.89, 95%CI 1.18–3.02], respectively. Children who ever suffered side-effects following immunization and those who travelled long distances to reach the vaccination centre had reduced odds of full uptake of malaria vaccine [OR: 0.35, 95%CI 0.06–0.25 and OR: 0.30, 95%CI 0.03–0.39] respectively. Only 17% (n = 65) of mothers/caregivers knew the correct schedule for vaccination and 38.5% (n = 158) knew the correct number of doses a child was to receive.

**Conclusion:**

Only RTS,S dose 1 and 2 uptake met WHO coverage targets. Mothers/caregivers had low level of information regarding malaria vaccine, especially on numbers of doses to be received and dosing schedule. The primary modifiable factor influencing vaccine uptake was mother/caregiver knowledge about the vaccine. Thus, to increase the uptake Nsanje District Health Directorate should strengthen communities’ education about malaria vaccine. Programmes to strengthen mother/caregiver knowledge should be included in scale-up of the vaccine in Malawi and across sub-Saharan Africa.

## Background

Malaria continues to pose a significant global health challenge. Globally, an estimated 249 million cases of malaria occurred in year 2022 which was 2 million more cases than in 2021 [1]. Sub-Saharan African countries facing the hardest hit contributing 93.6% (233 million) of total malaria cases and 95.4% (580,000) of total malaria deaths [1]. In Africa, about 78.1% (453,000) of the total deaths in 2022 were children below 5 years [1]. Malawi is among the 15 countries with the highest burden of malaria reporting over 4 million estimated malaria cases were reported in 2022 [1].

To control malaria, the National Malaria Control Programme (NMCP) within the Malawi Ministry of Health (MOH) currently supports the following interventions: long-lasting insecticidal nets (LLINs), prompt diagnosis with effective treatment with artemisinin-based combination therapy (ACT), and indoor residual spraying (IRS). The RTS,S/AS01 malaria vaccine is a new addition to malaria control tools [2]. In 2021, the World Health Organization (WHO) recommended RTS,S/AS01 for children at risk of malaria in sub-Saharan African regions of moderate to high malaria transmission [3]. The successful deployment of a malaria vaccine could substantially reduce the burden of malaria-related morbidity and mortality in under five children 4–6 . However, vaccines cannot achieve their anticipated benefits if the uptake is low. It is estimated that 1 out of every 5 children do not receive basic vaccines which contributes to more than 30 million under five years children suffering from Vaccine Preventable Disease (VPDs) each year [5]. For instance, the 2023 measles outbreak in South Africa was caused due to low coverage of measles vaccine [7]. Similarly, there was an outbreak of Polio in Cameroon due to low coverage of Oral Polio Vaccine (OPV) [8], low vaccine coverage has contributed to infectious disease outbreaks in vulnerable population [9]. Vaccine hesitancy also contributes to low vaccination coverage in many Africa countries [10].

In 2019, a Phase IV implementation study of the RTS,S/AS01 vaccine delivered through routine EPI platforms was conducted in Malawi. The four required doses of malaria vaccine were delivered at 5, 6, 7, and 22 months. The implementation study took place in 11 districts, including Nsanje. The effectiveness and impact of malaria vaccine relies not only on its introduction to the country but also on its widespread acceptance and uptake. This research study aimed to quantify the uptake of malaria vaccine in Nsanje district and investigate the factors associated with malaria vaccine uptake, including sociodemographic, mother/caregiver-related factors, and health care system factors. Identification of these factors may help to develop approaches to accelerate high levels of uptake of malaria vaccines, thereby advancing the global agenda towards malaria eradication.

All the 4 contacts for malaria vaccine are new and are not given with any other EPI interventions (Table 1). The only other vaccine that is given in the second year of life is measles and rubella vaccine dose 2, which is given at 15 months.

**Table 1:** Vaccination schedule in Malawi EPI (with malaria vaccine added)

Vaccine	Description	Schedule
BCG	Bacillus Calmette–Guérin (dose)	At birth or first contact
OPV0	Oral polio vaccine 0 (dose)	At birth to 2 weeks
Rotavirus	Rotavirus vaccine (two doses	6 and 10 weeks
Pentavalent	Diphtheria and tetanus and pertussis and *Haemophilus influenzae* and hepatitis B (three doses)	6, 10 and 14 weeks
OPV	Oral Polio Vaccine (three doses)	6, 10 and 14 weeks
Pneumo_conj	Pneumococcal conjugate vaccine (three doses)	6, 10 and 14 weeks
MV	Malaria vaccine/RTS,S/AS01 (four doses)	5, 6, 7 and 22 months
MR	Measles and rubella vaccine (Two doses)	9 and 15 months

According to the WHO, it is recommended that the first dose of RTS,S vaccine should be received at 5 months, with the successive doses being received at 1 month apart and the 4th dose to be received at 15–18 months after the third dose. However, the WHO also states that vaccination programmes may choose to give the first dose at a later or slightly earlier age based on operational considerations. At 11 months, is the latest month a child can receive the first dose of malaria vaccine and at 36 months is the latest month for a child to receive dose 4.

## Methods

### Study area

Nsanje district is located in the southern region of Malawi. It is situated at the tip of the country along latitude 16°45′00″S and longitude 35°10′00″E. The district is a flatland in the lower Shire valley covering 1,942 square kilometres with an estimated population of 299,168 11, 12 .The district has 23 health facilities (3 hospitals, 12 health centres and 8 health posts) which are divided into five clusters for health administration purposes. The malaria vaccine was implemented in four out of the five clusters. This study was conducted in the catchment areas of health facilities within each implementation cluster: Mlolo cluster (Mlolo, Trinity, Masenjere, Makhanga, Sankhulani and Mchacha), Kalemba cluster (Kalemba, Phokera, Sorgin, Misamvu, Kanyimbi), Tengani cluster (Tengani, Nyamithuthu and Mkango) and Boma cluster (Nsanje district Hospital and Chididi) [12]. Cluster sampling was performed.

### Study design

This was a cross-sectional study utilizing structured questionnaires together with checklists to collect data on the uptake of malaria vaccine and factors that influence uptake. Data on child vaccination status were obtained by reviewing their health passport. Mothers/caregivers were asked demographic questions about themselves and their children and also about factors that were associated with the uptake of the malaria vaccine.

### Study population

In February 2023, names of the mothers/caregivers of children 24–36 months old were extracted from village registers with the help of Health Surveillance Assistants. Children in this age group were eligible to have received all four doses of the vaccine. After all the names were extracted from the village registers, the participants were selected using stratified random sampling. Mothers/caregivers of the selected children were contacted and eligibility assessment was conducted. The eligibility criteria were: a mother/caregiver responsible for the selected child aged 24–36 months by the time of data collection, and the child was a permanent resident of Nsanje district by birth.

### Sample size calculation

Using Cochrane’s formula to estimate proportion of children receiving vaccine (prior estimate of dose 4 = 60%) with a margin of error of 5% assuming a normal distribution of the margin of error, the minimum sample was calculated to be 369 participants. After adjustment for a 10% non-response rate and rounding the target sample size was 410.

The total population in the four target clusters is 245,620 including an estimated 12,281 mothers/caregivers. Stratified sampling technique proportionate to size of the cluster population was used in selecting participants for the study. Table 2 shows the sample proportions.

**Table 2:** Sample size determination by cluster (proportionate to size)

Cluster	Total population	Target population	Proportion	Sample
Mlolo	74,606	3894	32	130
Boma	47,085	2336	19	78
Tengani	58,975	2906	24	97
Kalemba	64,954	3145	26	105
Total	245,620	12,281	100	410

### Sampling individual participants

To sample individual respondents in this study, firstly, systematic sampling technique was used to selected respondents from each sub-cluster. The Village Health Registers were used as source of the names for mothers/caregivers. The names of all mothers/caregivers who met the eligibility criteria in each cluster were numbered and written down, thus forming the sampling frame. A formula was used to determine a sampling interval from each cluster. The formula that was used was i = N/n where i was the sampling interval, N was the total number of eligible mothers/caregivers in the sampling frame whereas n was sample size of the cluster. The names and the villages of the mothers/caregivers counting from one to the sampling frame were written on a piece of paper, folded, mixed thoroughly and put in a box then a simple random sampling technique was used to select the first sample. After the first sample was drawn, the initial list that was written down was used to select the subsequent participant. The subsequent participants were selected by adding the sampling interval to the number of the initial sample until the required samples were drawn for that cluster.

### Data collection

Questionnaires were administered by research assistants to the study participants. Questions included mothers/caregiver socio-demographic characteristics, child factors (e.g. history of vaccine adverse reactions), community level factors and health care system factors. Information on vaccine uptake and timeliness was extracted from the health passport of the child. For mothers/caregivers who did not have health passports for the children, data was recorded using recall history. The questions were adopted from previous validated questionnaires used in Malaria Indicator surveys and Demographic and Health surveys. Mothers/caregivers were contacted in their homes depending on when they were available to respond to the questionnaire; interviews were not conducted at health facilities or at the vaccination point. Ethical approval was obtained from Jaramogi Oginga Odinga University of Science and Technology, approval number ERC 37/04/23-5/05 and from Malawi National Health Sciences Research Committee, protocol number 23/02/3167. Informed consent was sought from each study participant.

### Data analysis

Data from paper-based questionnaires were entered in Microsoft Excel by a single study team member. A second team member double-checked data entry by comparing the questionnaires and the data entered in Microsoft Excel. The cleaned data set was imported to STATA version 16 for analysis. Descriptive statistics were calculated for binary, categorical, and continuous variables. Logistic regression was used to evaluate the association between the independent variables (for example socio-demographic) and the level of malaria vaccine uptake (dependent variable). Malaria vaccine uptake was divided into three categories: *no uptake* (child has not received any dose of malaria vaccine), *partial uptake* (child has received first, second or third dose) and *full uptake* (child has received all four doses). After the univariate analysis, a multivariate analysis was performed on those independent variables with significant p-values of 0.05 in the first stage. The binary regression involved the comparing between full malaria vaccine uptake against partial uptake and no uptake. Final model selection was based on having the lowest Akaike’s Information Criteria (AIC).

## Results

### Socio-demographics characteristics of mothers/caregiver and their children

A total of 410 mothers/caregivers with children aged 24 to 36 months participated in this study. Participants were most commonly married females (79.8%, n = 327) who were 20–29 years old (54.2%, n = 222), self-employed (58.8%, n = 241), Christians (95.1%, n = 390), and were most often parents as opposed to caregivers (Table 3). Education level varied with more than half having no formal education (22%, n = 90) or only primary school (36%, n = 149). Most had at least four antenatal care visits during pregnancy (56.2%, n = 168). Out of the 410 participants, 82.4% (338) had their children’s health passports present whereas 17.6% (72) had no health passports for their children. The median age of the children whose data was collected was 29 months (IQR 26–33), half were male, and almost all were delivered at a health facility (98.8%, n = 405) (Table 4).

**Table 3:** Socio-demographic characteristics of mothers/caregivers in Nsanje district, 2023

Characteristic (n = 410)	Category	n	Proportion %
Age group (years)	Less than 20	31	7.6
	20–29	222	54.2
	30–39	125	30.5
	40 and above	32	7.8
Sex	Female	374	91.2
	Male	36	8.8
Education level	No education	90	22
	Primary	149	36.3
	Secondary	167	40.7
	Tertiary	4	1
Marital status	Single	44	10.7
	Married	327	79.8
	Divorced	26	6.3
	Widowed	13	3.2
Religion	Christianity	390	95.1
	Islam	19	4.6
	Traditionalist	1	0.2
Occupation	Unemployed	67	16.3
	Self-employed	241	58.8
	Farmer	89	21.7
	Civil servant	13	3.2
Parity	1 to 3	254	62
	4 and above	156	38
Antenatal care visit	1 to 3	131	43.8
	4 and above	168	56.2

**Table 4:** Distribution of characteristics of children who participated in the study in Nsanje district, 2023

Characteristic (n = 410)	Category	n	Proportion %
Age group (months)	24–27	176	42.9
	28–31	85	20.7
	32–36	149	36.3
Sex	Female	199	48.5
	Male	211	51.5
Delivery place	Health facility	405	98.8
	Home	4	1
	Don’t know	1	0.2

### Uptake of malaria vaccine

**Fig. 1:**
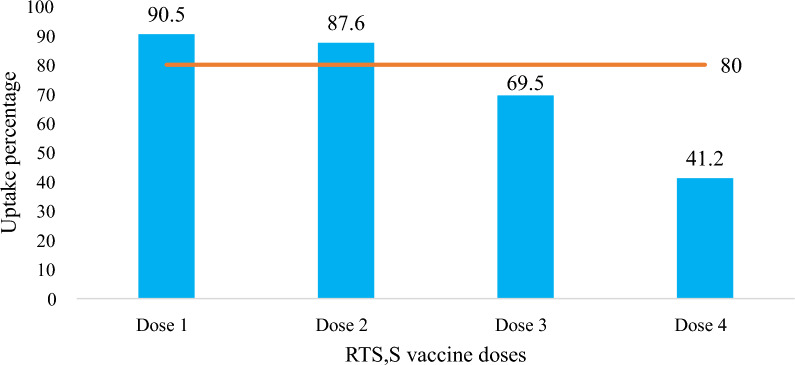
Uptake of malaria vaccine among children aged 24–36 months in Nsanje district. Blue bars: uptake (%), Orange bar: recommended target

The crude uptake was used for this study (vaccinations from health passport plus mothers/caregivers recall). Out of the 410 children, 9.5% of children did not receive any doses of the malaria vaccine. Among those who received the vaccine, coverage was relatively high for dose 1 and 2 (90.5% and 87.6%, respectively), but declined for dose 3 and 4 (69.5% and 41.2%, respectively) (Fig. 1). Thus, the levels of malaria vaccine uptake were 9.5% (n = 39) for *no uptake*, 49.3% (n = 202) for *partial uptake* and *full uptake* 41.2% (n = 169) (Table 5). Children of the 72 mothers/caregivers who had no health passports of their children had the following levels of uptake of malaria vaccine (no uptake, 33.3%, n = 24), while 40.3% (n = 29) had partial uptake, and 26.4% (n = 19) had taken all the doses of malaria vaccine (full uptake).

**Table 5:** Malaria vaccine uptake levels for children age 24–36 months in Nsanje district, 2023

Health passport plus mothers/caregiver recall n = 410			Health passport only n = 338	
Level of uptake	n	%	n	%
No uptake	39	9.5	15	4.4
Partial uptake	202	49.3	173	51.2
Full uptake	169	41.2	150	44.4
RTSS 1				
Yes	371	90.5	323	95.6
No	39	9.5	15	4.4
RTSS 2				
Yes	359	87.6	311	92.0
No	51	12.4	27	8.0
RTSS 3				
Yes	285	69.5	272	80.5
No	125	30.5	66	19.5
RTSS 4				
Yes	169	41.2	150	44.4
No	241	58.8	188	55.6

### Reasons for incomplete vaccination

Among the 39 participants whose child did not receive any dose of malaria vaccine, 26 (67%) did not know their child was eligible, nine (23%) said their religious belief prohibited them from taking the vaccine, and four (10.3%) made a personal decision to refuse the vaccine. Out of the 202 participants who had partial uptake of the vaccine, 70.4% did not know the next date when the vaccination was due and 15 (4%) were not comfortable with issues surrounding vaccines. In total 28 participants reported vaccine hesitancy with their reasons being complete refusal (n = 4), religious reasons (n = 9) and not comfortable with issues surrounding vaccine (n = 15) leading to no or partial uptake (Table 6).

**Table 6:** Reasons for malaria vaccine partial or non-uptake in Nsanje district, 2023

Reasons for not taking any of the doses	n	%
Did not know the child was eligible	26	66.7
Religious beliefs	9	23
Personal decision to refuse vaccine	4	10.3
Reason for not taking all the doses		
Did not know when next one was due	261	70.4
Was not around	81	21.8
Child was sick	14	3.8
Not comfortable with issues surrounding vaccine	15	4

### Mother/caregiver and child characteristics associated with vaccine uptake

Mothers/caregivers who had secondary or higher education had increased odds of having full uptake of malaria vaccine compared to their counterparts (OR = 2.43, 95% CI 1.43–4.12, p = 0.001). Having attended four or more antenatal visits was associated with full uptake of malaria vaccine (OR = 1.89, 95% CI 1.18–3.02, p = 0.008). However, there was no statistically significant association between sex, religion, occupation of the mother/caregiver and full uptake of malaria vaccine. Children aged 32 to 36 months had increased odds of full malaria vaccine uptake compared to those aged 24 to 27 months (OR = 1.72, 95% CI 1.11–2.69, p = 0.008) whereas sex and place of delivery was not statistically associated.

General knowledge about the malaria vaccine was associated with increased vaccination rates; mothers/caregivers who had heard about malaria vaccine prior this study had increased odds of full malaria vaccine uptake compared to those who never heard about it (OR = 4.47, 95%CI 1.29–15.41). Those who received messaging about the malaria vaccine from under 5 clinic had increased odds to having full malaria vaccine uptake compare to those who learned about the vaccine from the radio (OR = 3.15, 95% CI 1.22–8.11, p  = 0.018). However, detailed knowledge about the vaccine, for example, knowing the number of malaria doses to be received and the specific age at which those doses should be received, was not associated with full uptake of malaria vaccine.

Children who ever suffered side effects following immunization were associated with reduction in full uptake of malaria vaccine (OR = 0.36, 95% CI 0.24–0.54, p < 0.001).

Mode of transport was associated with full uptake of malaria vaccine. Those mothers/caregivers who used motorbikes/bikes to go the vaccination point had increased odds (OR = 2.79, 95% CI 1.50–5.18, p = 0.001).

Having heard negative rumours about the malaria vaccine, for example that children were being used for experiments or that the vaccine will affect child development, reduced the odds of full uptake by 25% (OR = 0.25, 95% CI 0.14–044, p ≤ 0.001). Mothers/caregiver who had no problem with the introduction of malaria vaccine had increased odds to full uptake of the vaccine (OR = 3.47, 95% CI 1.29–9.39, p = 0.014).

### Multivariate analysis of associations between full uptake of malaria vaccine and mother/caregiver and child factors

Multivariate logistic regression showed that the odds of malaria vaccine uptake was 26.56 times to those who ever heard of malaria vaccine than those who did not. Further, it showered that distance to vaccination point reduced the odds of full uptake by 24% whereas child ever suffered side effects following immunization reduced the odds by 23%. Table 7 below shows the details of multivariate logistic regression.

**Table 7:** Predictors of malaria vaccine uptake among children aged 24–36 months in Nsanje district, 2023

	OR	95% CI	P value	aOR	95% CI	P value
Education level	1.77	1.35–2.32	< 0.001	1.49	1.01–2.14	0.03
No education	1			1		
Primary	0.64	0.37–1.13	0.125	0.46	0.21–1.01	0.052
Secondary and above	2.43	1.43–4.12	0.001	1.73	0.83–3.57	0.141
Antenatal clinic visit	1.89	1.18–3.02	0.008	1.70	0.96–3.02	0.069
1 to 3	1			1		
4 and above	1.89	1.18–3.02	0.008	1.66	0.93–2.99	0.087
Distance to vaccination point	0.26	0.17–0.40	< 0.001	0.24	0.14–0.43	< 0.001
Less than 30 min	1			1		
More than 30 min	0.26	0.17–0.40	< 0.001	0.23	0.13–0.42	< 0.001
Attended vaccination site and failed to vaccinate a child	2.66	1.65–4.28	< 0.001	4.65	2.21–9.78	< 0.001
No	1			1		
Yes	2.66	1.65–4.28	< 0.001	4.81	2.28–10.15	< 0.001
Mode of transport to vaccination point	1.67	1.23–2.27	0.001	2.06	1.36–3.13	0.001
Walking	1			1		
Commercial (motorbike/bike)	1.62	0.10–26.12	0.734	2.37	0.10–56.64	0.595
Personal vehicle(car/bike/motorbike)	2.79	1.50–5.18	0.001	3.86	1.64–9.13	0.002
Ever heard of malaria vaccine	4.47	1.29–15.41	0.018	22.17	2.25–218.27	0.008
No	1					
Yes	4.47	1.29–15.41	0.018	26.56	2.64–266.97	0.005
Experience with side effects following immunization	0.36	0.24–0.54	< 0.001	0.23	0.13–0.40	< 0.001
No	1					
Yes	0.36	0.24–0.54	< 0.001	0.23	0.13–0.41	< 0.001

## Discussion

This study found that only the uptake of the first and the second doses of the RTS,S malaria vaccine met target of coverage for childhood vaccines set by the WHO [13]. Coverage for the subsequent doses fell below the target with the 4th dose reaching few than half of eligible children. This result means that the malaria vaccine cannot meet its intended purpose of averting childhood malaria morbidity and mortality unless its uptake for full vaccination can be improved. Decreasing coverage after the first dose of a multi-dose vaccine is common and has been reported for RTS,S in Ghana [14] as well as other childhood vaccine studies on vaccine uptake^15^–^17^ .

The high coverage of RTS,S doses 1 and 2 could have been achieved due to of the campaign conducted during the launch of the vaccine in the routine vaccination system in Nsanje district. This possibly created a lot of demand for the vaccine and it made the communities aware of the vaccine. The district health directorate created demand through risk communication during community engagement. Later after the launch campaigns, the demand could have been reducing which could lead to reduction of the subsequent doses.

The data for coverages of other vaccines offered in the district the same period when this study was conducted was high. Then coverage for BCG was at 99.5%, MR 1 was at 97%, MR 2 was at 92%, Rota 1 was at 98.6% and for Rota 2 was at 94.3%. No vaccine was below 80% whether it was administered once or had several numbers of doses. This showed that only the malaria vaccine had the lowest coverage for full uptake.

Knowledge of the mothers/caregivers on the childhood vaccines, ages at which those vaccines are received and the number of vaccines doses a child should receive to be fully vaccinated is important in order to increase the uptake levels of the vaccine. Although the majority of the mothers/caregivers had ever heard about malaria vaccine, only a few knew the vaccination schedule and number of doses to be received for a child to be fully vaccinated. This poor knowledge could have contributed to the reduction of subsequent doses observed in this study. This indicated that health education and promotion on malaria vaccine is not adequately done in Nsanje district. Similarly, a study conducted by Biset et al*.* [16] found that low knowledge about childhood vaccine was associated negatively with full vaccination coverage. Most mothers/caregivers relied on the community health volunteer or the Health Surveillance Assistant to remind them about the next day of vaccination hence there was no association between having knowledge on vaccine schedule and ages with full uptake of malaria vaccine. However, it is very important that the mothers/caregivers should know the ages and the vaccination schedule in cases whereby the community health volunteer or Health Surveillance Assistant did not remind them about the next visit day, they should be able to remember by themselves. In doing so, the coverages could be high. In a study conducted by Victoria et al*.* [18], in Ghana, concluded that health education is important because fears and concerns about malaria vaccine are addressed. Addressing the mothers/caregivers concerns through health education may enable the mothers/caregivers to encourage other mothers/caregivers in their communities to get their children vaccinated hence increasing the vaccine coverage. Additionally, some systematic reviews conducted in Africa on childhood vaccination also found that full uptake of childhood vaccines was influenced by mothers knowledge on vaccines^19^, ^20^ .

In this study, few children did not take any malaria dose. The main reason was that their mother/caregiver not knowing that their children were eligible while some did not take any dose of malaria vaccine because of religious beliefs. In Nsanje district there are certain religions that prohibits its member to go to the hospital or access any other health services. Since mothers/caregivers from these religions are likely not be found at under 5 clinic to learn the importance of malaria vaccines, even if they are willing to vaccinate their children, their religious leader will prevent them from accessing the health services. This was also evidenced in a study conducted by Adeyanju et al*.* [21] in Malawi, which reported that religious groupings, for example Zion and Apostolic faith, were prohibiting their members from visiting the hospital and accessing vaccines.

A majority of the children in this study had no or partial uptake of malaria vaccine. The main reason for no/partial uptake being low levels of knowledge and awareness on next visit date and knowing if their child was eligible. This finding is similar to reports by Price et al*.* [22] which reported that in first three implementing countries of malaria vaccine, information barrier contributed to no or partial uptake of malaria vaccine. Additionally, in Kenya, a recent study by Hoyt et al*.* [23] found that lack of awareness on malaria vaccine was a factor that led to lower coverage of malaria vaccine. Another study by Yeboah et al*.* [24] in Ghana recommended that mothers should understand the importance of their children getting the vaccine even during their second year of life, to help increase the uptake of dose 4 of malaria vaccine.

Furthermore, this study showed that the education level of mothers/caregivers was associated with full uptake of malaria vaccine. Mothers/caregivers who had secondary education and above managed to fully vaccinate their children with malaria vaccine. The high uptake of malaria vaccine by those mothers/caregiver that are more educated is because as they can easily understand the importance of malaria vaccine to their children, but also they could have greater access to information regarding malaria vaccines and other vaccines in general. This finding is consistent with the findings from a study conducted in Malawi [25]. In additional, a study conducted in Burkina Faso reported that level of education was a determinant in the uptake of childhood vaccines [26]. A systematic review conducted in Sub-Saharan Africa by Tekle et al*.* [27] and another study conducted by Touray et al*.* [27] found that level of education of a mother/caregiver was associated with full uptake of childhood vaccine.

Number of antenatal visits was a factor affecting full uptake of malaria vaccine. The children whose mothers/caregivers went for ante natal clinic 4 times or more had increased odds of getting fully vaccinated. This could be due to their health seeking behavior but also because they could have heard about the introduction of malaria vaccine at ANC and being told the importance of vaccinating their children. Similar results were reported in studies conducted by in Malawi,  Ghana and Kenya [23–25].

This study showed that mothers/caregivers whose children ever suffered side effects following immunization had decreased odds of completing all the four doses of malaria vaccine. These mothers/caregivers could have been afraid of taking their children for vaccination in fear of the side effects. Studies conducted in Nigeria, Burkina Faso and Ethiopia also reported side effects following immunization affected the uptake of childhood vaccines [21, 25, 26]. Since malaria vaccine was being newly introduced in Nsanje district mothers/caregivers could think that the vaccine may have worst adverse effects after immunization hence hesitating in the uptake. This could have contributed to the low coverage of fully vaccinated children with malaria vaccine.

Mothers/caregivers who were living near the vaccination point had increased odds in getting their children receiving all the doses than those mothers who were living far. Similar results were observed in studies conducted in Malawi . Three systematic reviews conducted in Ethiopia, Nigeria and in sub-Saharan African systematic also found that distance to the vaccination site was a determinant for full uptake of childhood vaccines^16^, ^20^, ^28^ Furthermore, mode of transport was found to be a significant factor associated with full uptake of malaria vaccine. This study observed that those mothers/caregivers who used commercial motorbikes or bikes were finding it easy to reach the vaccination points hence most of them had their children full vaccinated. Similar findings were reported in a study conducted in Togo [29].

The study found that having attended vaccination site and failed to vaccinate the child was significantly associated with malaria vaccine uptake. The reasons for mothers/caregivers not vaccinating their child while already at the vaccination site could be vaccine stock-outs, cancellation of the vaccination clinic, arriving at the clinic late while it has already been closed. This could have made some mothers/caregivers not to go to the vaccination site again as they think they may just waste their time to go to the vaccination site and never vaccinate their children hence leading to low coverage of malaria vaccine. Similarly, Lee et al*.* [30] found that vaccine stock-outs were significantly associated with low coverage of vaccines. Additionally, a study conducted in Ethiopia in 2021 reported that mothers/caregivers unsatisfied with health care workers was a determinant for incomplete childhood vaccination [31].

## Limitations of the study

Some of the mothers/caregivers had no health passports for their children and consequently the researcher only relied on the word of mouth to re-call some information. This study did not collect qualitative data using Focus Group Discussions (FGDs), this would have thrown more light on the health system factors affecting the uptake of malaria vaccine it could also have given more understanding on perceptions, experiences and challenges faced by mothers/caregivers getting their children to receive malaria vaccine.

## Conclusion

In order to reach WHO vaccine targets and increase the effectiveness of the malaria vaccine, district and national level agencies, e.g. Nsanje Directorate of Health and Social Services and Malawi Ministry of Health, should intensify and sustain information, education and communication in the communities about the malaria vaccine. Engaging religious leaders may also enhance these messages.

## Data Availability

Relevant datasets for this study are availability from the corresponding author upon request.

## Abbreviations

AEFI: Adverse event following immunization
ANC: Ante-natal care
EPI: Expanded programme on immunization
LLIN: Long lasting insecticidal nets
MOH: Ministry of Health
NMCP: National Malaria Control Programme
PBO: Piperonyl butoxide
RTS,S: Malaria vaccine
VDP: Vaccine preventable diseases
WHO: World Health Organization
